# Weight biases, body image and obesity risk knowledge in the groups of nursing students from Poland and Nigeria

**DOI:** 10.1038/s41598-024-54904-1

**Published:** 2024-02-22

**Authors:** Wojciech Styk, Marzena Samardakiewicz, Szymon Zmorzynski

**Affiliations:** 1 Department of Psychology, Medical University of Lublin, Lublin, Poland; 2 The Academy of Zamość, Zamość, Poland; 3 Academic Laboratory of Psychological Tests, Medical University of Lublin, Lublin, Poland

**Keywords:** Weight biases, Crosscultural, BMI, Obesity, Medical students, Fat phobia, Psychology, Human behaviour

## Abstract

Each population may have its own specific characteristics and cultural differences, which can affect perceptions of one's body, obesity, and the development of weight-related biases. The goal of our study is to (I) examine weight biases among incoming nursing students from two distinct cultures; (II) determine whether the cultural differences may be reflected in the levels of fat phobia, attitudes, and behaviors related to overweight and obesity; (III) adapt the Fat Phobia Scale and translate it into Polish. The study includes 119 Nigerian students and 120 Polish students. The following tools are used—ORK-10, ATOP, BAOP, BES and FPh. The results indicates that Nigerian students have significantly (*p* < 0.01) less knowledge about the risks associated with overweight and obesity. In contrast, they have a significantly (*p* < 0.01) more positive body image than the Polish students. Among Nigerian students, men have more positive body image in comparison to women (*p* = 0.01). An inverse relationship is observed in the group of Polish students, among whom women had a more positive body image than men did (*p* = 0.01). There are no statistically significant differences in fatphobic attitudes among the studied groups. It has been observed, that culture may be related to weight biases.

## Introduction

Obesity is a major health problem worldwide. According to the World Health Organization (WHO), in 2016, more than 1.9 billion adults worldwide were overweight, and 650 million were obese. The increase in the number of obese and overweight people is particularly evident in developed countries, where lifestyles and diets favor obesity^1^. Obesity increases the risk of diseases such as diabetes mellitus type 2, cardiovascular disease, hypertension, and cancer. Diseases leading to obesity are important factors affecting health status and represent a significant cost to health systems and societies. The fight against obesity is a major health challenge worldwide. It requires commitment from the government, the private sector and society as a broader unit^2^. Obesity indicators in Europe and Africa vary and depend on many factors, such as standard of living, lifestyle, diet, and culture. Overall, the incidence of obesity is greater in Europe than in Africa. According to 2016 WHO data, the obesity prevalence in Europe was 23.3%, while in Africa, it was 8.7%. However, it is worth noting that the frequency of obesity in different countries and regions can vary significantly^1,3^. Research consistently shows that obesity prejudice has a detrimental impact on the prevention and treatment of obesity. Weight stigma is linked to maladaptive eating behaviors, reduced physical activity, and physiological stress responses, all of which can exacerbate obesity^4^. Stigmatizing attitudes of health care personnel toward people with obesity have a negative impact on adults, children, and adolescents with obesity. This manifests itself in reduced physical activity, social isolation and decreased access to health care^5,6^. Research on this phenomenon indicates that people with obesity face various types of stigmas, including direct, environmental and indirect stigmas, in their daily lives. This can cause barriers related to a lack of willingness to be physically active^7^. Weight-based stigma can also vary by sex, sexual orientation, age, and other social and cultural factors. Notably, weight-based stigma can lead to harmful consequences for the mental and physical health of affected individuals and can also contribute to social inequality and discrimination^8^.

Studies show that even professionals who treat eating disorders have negative attitudes and stereotypes about obese patients. Additionally, weight-related terms used by health professionals, such as "morbidly obese," "fat" and "obese," were rated as the most undesirable, stigmatizing and blaming language^9,10^. These findings suggest that negative beliefs about overweight and obese patients still exist among healthcare professionals. Attitudes and views of health care professionals related to weight can have a negative impact on patient-provider relationships and the quality of provided care. Weight stigma among health care providers reduces the quality of care received by obese patients^4,10^. In response to these issues, large-scale interventions are being introduced with the goal of reducing weight bias in students and health professionals. According to previous research, these conditions result in a small to moderate decrease in obesity-related biases^11^. Research in this area indicates that patients generally prefer neutral terminology when discussing weight, and words such as "obese" and "fat" are the least acceptable, especially in provider-patient conversations about their weight^9^.

Research papers on weight biases indicate that they are common at the study stage. Health science students had greater weight biases than nursing and medical students. Another study revealed that medical students had negative attitudes toward overweight people and ascribed personal responsibility for obesity to these individuals. In contrast, physiotherapy students had negative attitudes when working with obese patients and believed that obesity was caused by patient behavior. A study of Polish nutrition students revealed prejudice and negative attitudes toward obese patients, especially toward obese women^12–14^. These findings suggest that weight biases and negative attitudes toward overweight and obesity are common among health science students and highlight the need for interventions to resolve these biases during education^15,16^. One way to overcome negative prejudices is through training and education. Studies show that reliable knowledge about obesity risk is associated with less negative attitudes toward overweight and people with obesity. Anti-discrimination training among nursing students increases knowledge and decreases prejudice and discrimination against overweight or obese individuals^17,18^.

However, it is important to remember that each population may have its own specific characteristics and cultural differences. People from various regions of the world may have completely different perceptions of obesity and weight biases. Different cultures have different norms and values related to body weight, which affects what behaviors and traits are considered desirable or undesirable. For example, in Western culture, thinness is often considered an attractive trait, while overweight and obesity can be considered negative and stigmatized^4^. However, there are also cultures where a body with a higher weight is considered desirable, for example, in some African cultures such as the Igbo and Yoruba^19^. In such cultures, individuals who have higher weights may be treated with more respect and considered more attractive than thin people^9^. Therefore, studies conducted on different populations and fields of study may yield different results.

Taking into account above we decide to analyse weight biases among newly enrolled nursing students from two different African and European cultures. In order to achieve this goal we check whether the differences regarding both obesity epidemiology and culture can reflect in levels of fat phobia, attitudes, and behaviors regarding overweight and obesity and whether these relationships are influenced by perceptions of one's body. An additional goal is to adapt and translate the Fat Phobia Scale into Polish.

## Results

### Analysis of variables in the studied groups

To visualize the differences between the analysed groups of students from Poland and Nigeria, the first step is to compare the mean values of the studied variables. The means, standard deviations and group sizes are presented in Table 1. The table also includes the results of the Mann‒Whitney test for mean comparisons. The effect size is given by the rank biserial correlation (*r*_*rb*_). The conducted analyses indicate statistically significant differences for most of the analysed variables. The only variables that do not differentiate the study groups are the age of the subjects and the Fat Phobia test score. The power effect of these correlations is strongest for the level of knowledge about obesity risk (ORK) and body image (BES). Nigerian students have significantly less knowledge about the risk of overweight and obesity. In contrast, they have a significantly more positive body image than do the Polish students. The average BMI of Nigerian students is greater in comparion to Polish students (Table 1). However, it should be noted that this value is within the threshold of being overweight.

**Table 1:** Differences in the mean values of the studied variables between a group of students from Nigeria (NGR) and Poland (PL).

	Group	M	SD	*p*	Effect size (*r*_*rb*_)
ORK	NGR	2.59	1.61	< 0.01	
	PL	5.20	1.45		− 0.75
ATOP	NGR	71.30	13.29	< 0.01	
	PL	77.60	9.41		− 031
BAOP	NGR	24.28	4.88	< 0.01	
	PL	26.79	3.77		− 0.32
FPh	NGR	3.09	0.91	0.34	
	PL	3.11	0.24		–
BES	NGR	126.25	20.68	< 0.01	
	PL	71.18	56.80		0.59
BMI	NGR	25.70	5.68	< 0.01	
	PL	23.78	3.56		0.20
AGE	NGR	25.24	5.67	0.05	
	PL	28.10	8.94		–

Mann‒Whitney test.

In the next step, we check whether there are differences in the analysed groups of students according to sex. Table 2 shows the results of the our analyses, with the determine effect size indicating statistically significant values. Among the students from Nigeria, there are differences in the level of knowledge regarding the risk of overweight and obesity; women have knowledge at higher level. Differences are also observed in attitudes toward overweight people and people with obesity. Male students have more positive attitudes toward overweight people and people with obesity. Among the Polish students, differences are also observed in attitudes regarding overweightness and obesity; however, in this group, more positive attitudes are represented by women than men, and these differences are characterized by the highest effect size. In both the Nigerian and Polish student groups, sex differences are also observed in body image. Among the students from Nigeria, men have a more positive body image. In contrast, in the Polish group, women have a significantly better image of their bodies.

**Table 2:** Differences in the mean values of variables by sex in the studied groups.

	SEX	NGR		*p*	PL		*p*
		M	SD	(r_rb_)	M	SD	(r_rb_)
ORK	F	2.76	1.59	0.03 (0.25)	5.07	1.44	0.20
	M	2.15	1.60		5.46	1.47	
ATOP	F	69.74	12.76	0.04 (− 0.24)	79.90	8.79	0.01 (0.44)
	M	75.36	13.97		72.82	8.95	
BAOP	F	23.80	4.71	0.11	27.25	3.68	0.09
	M	25.52	5.18		25.85	3.84	
FPh	F	3.11	0.91	0.65	3.09	0.24	0.25
	M	3.03	0.91		3.14	0.23	
BES	F	123.48	19.40	0.01 (− 0.29)	82.54	51.12	0.01 (0.29)
	M	133.48	22.42		46.95	61.28	
BMI	F	25.82	5.92	0.52	23.47	3.43	0.08
	M	25.40	5.08		24.42	3.76	
AGE	F	25.49	6.08	0.95	29.21	9.52	0.12
	M	24.61	4.46		25.79	7.17	

Mann‒Whitney test.

### Correlation analysis in a group of Nigerian students

A correlation analysis of variables in a group of students from Nigeria is also conducted. A statistically significant negative correlation between fat phobia and attitudes toward overweight people and people with obesity is observed for the studied group. Students with lower Fat Phobia scores have more positive attitudes toward overweight people and people with obesity. A negative association is also observed with body mass index. Increasing BMI is associated with decreasing fat phobia.

The analysis is also conducted with sex as a control variable. The association between the fat phobia level and attitudes toward people with obesity is found only in the male group, while the association with BMI is found only in the female group. The data are shown in Table 3. The observed correlations are mostly classified as weak or average.

**Table 3:** Correlations of studied variables among Nigerians.

	ORK			ATOP			BAOP			FPh			BES			BMI		
	All	Female	Male	All	Female	Male	All	Female	Male	All	Female	Male	All	Female	Male	All	Female	Male
ATOP	− 0.10	− 0.16	0.16	–	–	–												
BAOP	− 0.10	− 0.05	− 0.13	0.27**	0.19	0.37*	–	–	–									
FPh	− 0.10	− 0.03	− 0.31	− 0.27**	− 0.17	− 0.50**	− 0.16	− 0.08	− 0.34	–	–	–						
BES	0.02	0.08	0.01	0.10	0.01	0.16	− 0.05	− 0.09	− 0.08	0.01	0.04	− 0.05	–	–	–			
BMI	0.17	0.21	0.03	0.01	− 0.03	0.10	0.01	− 0.05	0.19	− 0.20*	− 0.22*	− 0.15	− 0.04	− 0.02	− 0.07	–	–	–
AGE	0.07	0.12	− 0.13	− 0.06	− 0.11	0.13	0.08	0.06	0.21	− 0.10	− 0.07	− 0.24	0.01	0.02	0.05	0.29**	0.31**	0.21

*p < .05, **p < .01, ***p < .001.

### Correlation analysis in a group of Polish students

In the next step, a correlation analysis is carried out for the group of students from Poland. A statistically significant negative correlation is observed between fat phobia and beliefs and attitudes about overweight people and people with obesity. Subjects with lower fat phobia scores have more positive beliefs about overweight or obesity. A negative association is also observed between the level of knowledge about obesity risk and body image. Participants who have a lower ORK scale score have a more positive body image. Moreover, body image is positively associated with positive beliefs about overweight people and people with obesity. A negative relationship is also observed between attitudes toward overweight people and people with obesity and body mass index (BMI). As BMI increased, attitudes toward overweight people and people with obesity become less positive. The strongest association of the studied variables is a positive relationship between the age of the subjects and body image. The analysis is also conducted with the controlled variable of sex. A relationship between knowledge of obesity risk and body image is found only in the female group. In the male group, a negative association is observed between knowledge of obesity risk and attitudes toward overweight people and people with obesity. There is also a positive association (in the meale group) between age and beliefs about overweight people and people with obesity and about body image. The observed correlations are classified as weak to average. The data are shown in Table 4.

**Table 4:** Correlations of studied variables among Poles.

	ORK			ATOP			BAOP			FPh			BES			BMI		
	All	Female	Male	All	Female	Male	All	Female	Male	All	Female	Male	All	Female	Male	All	Female	Male
ATOP	− 0.15	− 0.01	− 0.34*	–	–	–												
BAOP	− 0.16	− 0.09	− 0.24	0.39***	0.24*	0.58***	–	–	–									
FPh	0.03	0.04	− 0.02	− 0.13*	− 0.06	− 0.20*	− 0.28**	− 0.25*	− 0.30	–	–	–						
BES	− 0.26**	− 0.22*	− 0.27	0.21*	0.07	0.19	0.08	− 0.06	0.17	0.13	0.19	0.15	–	–	–			
BMI	0.07	0.15	− 0.11	− 0.20*	− 0.23*	− 0.05	0.02	− 0.01	0.16	− 0.14	− 0.14	− 0.20	− 0.05	− 0.12	0.16	–	–	–
AGE	0.02	0.12	− 0.16	0.13	− 0.01	0.30	0.12	0.01	0.34*	− 0.07	− 0.01	− 0.16	0.40***	0.32**	0.50**	0.11	0.10	0.22

*p < .05, **p < .01, ***p < .001.

### Comparison of correlations across studied groups

The correlation analyses of the studied variables in the studied groups are presented in Sections “Procedure” and “Tools”. The results show that the studied groups have different characteristics. To compare the groups, we check whether the correlations that occurr differ significantly. For this purpose, the collected data are compared using the Z-Fisher test. The results are summarized in Table 5. The comparison of correlations show no statistically significant differences. It should be noted that the studied groups differ in the presence or absence of statistically significant correlations, such as the occurrence of a negative correlation (r = − 0.26) between the ORK and the BES in the Poles group and the absence of such a correlation in the Nigerian students group.

**Table 5:** Results of the Z-Fishers test for statistically significant correlations.

Comparison	SEX	Spearmans’ rho		Z Fisher	
		PL	NGR	Z	p
ATOP-BAOP	ALL	0.39	0.27	1.030	0.15
ATOP-BAOP	M	0.58	0.37	1.109	0.13
ATOP-FPh	M	− 0.20	− 0.50	− 1.402	0.08

## Discussion

Here we report that Nigerian students have statistically significant less knowledge about the risks associated with overweight and obesity. In contrast, they have statistically significant more positive body image than the Polish students. Body image is the subjective evaluation of one’s own body, that is, the way an individual perceives his or her body. This includes general perceptions of the body’s appearance and proportions, as well as emotional and value aspects related to one’s body. Body image is part of self-esteem and affects an individual’s self-esteem, behaviour and mental health^20–22^. Civilization and access to social media can have a negative impact on individuals’ body image and can lead to disorders related to self-perception. Studies show that the popularity and ease of accessing photos and videos that depict an idealized body image can increase social pressure on individuals to have the same body^23–26^. The results of our study confirm these reports. Nigerian students, on average, present a significantly more positive body image than Polish students do, and the effect of this difference is strong. Nigeria is a country with a different culture from Poland but also with lower civilization development, including access to social media. Research indicates that exposure to idealized male Instagram photos, especially those depicting naked and muscular men, results in lower body satisfaction among men^27^. Cultural and media factors, as well as personal weight and sexual well-being, have influenced women's satisfaction with their buttocks in Nigeria, Germany, the US and Japan^28^. Exposure to body ideals in Western media has been linked to increased body dissatisfaction among Kenyan individuals, Kenyan Americans and African Americans^29^.

Cross-cultural studies of body image indicate that the body image of pregnant individuals differs from that of individuals in other countries. Polish women internalize Western beauty standards to a lesser extent than American women do. The pursuit of thinness is more prevalent among Polish girls and children from regions with low GDP (gross domestic product). In contrast, Polish men overestimate the importance of muscularity in attracting women^30–32^. Poland's younger women have a greater discrepancy between their actual and ideal body image, and mass media have a stronger influence on body image in younger women^33^. A study by Taylor et al. indicated that Polish women in Poland had greater weight discrepancy and lower body recognition than Polish female migrants and British white women, suggesting that civilization changes in Eastern Europe may place women in the region at relatively high risk of developing negative body image^34^.

It should also be remembered that high civilization quality can also affect body image through, among other things, access to low-quality foods. The study indicated that the development of civilization may lead to an increase in the consumption of high-calorie foods rich in fats, sugars, and salt while reducing the intake of fresh fruits and vegetables and healthy sources of protein and carbohydrates^35,36^. Increasing access to high-calorie foods easily available in stores, fast food bars or restaurants in highly civilized countries is causing the population to consume more calories, leading to increased obesity rates^37^. A study assessing the relationship between eating habits and perceived body image among Polish adolescents and young adults indicated that more university students were obese or overweight than high school students were, and approximately ¾ of the obese respondents were not accepting of their bodies and had made attempts to reduce weight in the past^38^.

Our results indicate that despite having a more positive body image, Nigerian students have, on average, a higher BMI than Polish students do; these students are classified as overweight according to WHO guidelines. As the study indicates, there is a high prevalence of obesity in Nigeria. A 2013 study indicated that the prevalence of overweight individuals in Nigeria was 20.3–35.1%, while the prevalence of obesity was 8.1–22.2%^39^. In contrast, the 2022 study indicates that 39% of adult Nigerians are centrally obese, with more than half of adult women being centrally obese^40^.

The WHO highlights the need for public awareness of obesity and its health consequences, as well as the promotion of healthy lifestyles and physical activity^41^. In turn, cultural stereotypes of masculinity and cultural norms influence men's health-seeking behavior in Nigeria, which can affect their overall health^42^. Our results indicate that Nigerian students (in Poland) have significantly less knowledge regarding obesity risk than Polish students do. In our study, the strength of the effect of this relationship is very strong. These results confirm other work suggesting low levels of obesity knowledge among Nigerians. A 2020 study showed that most Nigerian undergraduate students had low obesity knowledge and inappropriate eating behaviors^43^. Other studies indicate that there is dissonance between perceived and measured body weight among adult Nigerians, with many overweight people and people with obesity incorrectly perceiving their weight category^44^. Conversely, the study indicates that many university students in Poland have poor eating habits, with more than 40% not eating vegetables at least once a day and more than half not eating fruit. Most medical students in Poland, especially men, do not lead healthy lifestyles, as many aspects of their lifestyles deviate significantly from the recommended guidelines. A higher BMI is associated with an increased risk of eating disorders in students. This was confirmed by studies in Hungary, Poland, and Ukraine^45–47^.

The global problem of overweight and obesity is related to weight prejudice, stereotypes, attitudes, and beliefs about overweight and obesity. Weight bias is a form of discrimination that involves treating people differently because of their weight or physical appearance. Negative weight bias is also a serious problem in health care^10^. Weight bias is found in various countries around the world, including Africa. On the African continent, the problems associated with obesity and weight bias are relatively less common than in other regions of the world, such as North America and Europe^7^. Traditionally, some African communities have a positive attitude toward fuller figures and regard them as symbols of physical well-being and health. However, with rapid urbanization and globalization, cultural trends are emerging that promote the slim figure as an ideal of beauty. As a result, problems related to weight bias and weight discrimination are developing in some parts of Africa, causing problems of inequality in access to health food, education and medical services, which can affect the health and fitness of people from different social classes and cause obesity. A 2014 study of students at the University of Nigeria showed that 36.7% of respondents were overweight or obese, and more than half of the subjects (52.6%) experienced some form of weight bias^48^. Our study indicates that there are no differences in terms of fatphobic attitudes. We find more positive beliefs and attitudes about overweight and obesity among Polish students. The study indicates a positive correlation between knowledge about obesity risk and positive attitudes toward overweight people and people with obesity^17^. This result can be explained by the greater knowledge of obesity risk among Polish students. Conversely, a study by Hebl et al. revealed that African American women are generally less likely to stigmatize obesity than Caucasian women are, but this could not be confirmed in our study^49^.

Every scientific study has limitations. The main limitation of this study is that it was conducted on a group of Nigerian students in Poland, not in their home environment. Although the study was conducted on students who were freshly enrolled in Poland, we cannot predict the impact of changes in the environment in which they live. Another limitation is the size of the groups, which prevents us from drawing more general conclusions at the level of general student groups or populations. Another limitation was that respondents self-reported weight and height on the questionnaire. In future research, we will increase the sample size, and weight and height measurements will be taken using properly validated instruments.

In our study we observe that culture origin may be related to weight bias. Moreover, self-compassion may be more strongly associated with perceptions of one's own body and canons of beauty. These findings can be used to develop prevention programs to counterweight bias with cultural differences in mind. Nigerian students have significantly less knowledge about the risks associated with overweight and obesity. Conversely, they have a significantly more positive body image than do the Polish students. Among Nigerian students, men have more positive body images than women do. An inverse relationship is observe in the group of Polish students, among whom women have a more positive body image than men do. There are no differences in fatphobic attitudes among the studied groups. Polish students have more positive attitudes and beliefs about obesity, but the strength of the effect of these relationships is low. Culture may be related to weight biases. However, it may be much more strongly related to self-perception and canons of beauty.

## Materials and methods

### Study sample characteristics

The research is conduct in Poland at universities educating both Polish and foreigners. Both groups of respondents are surveyed in Poland at the same nursing universities. The study participants are first year nursing students. Complete questionnaires are received from 119 students from Nigeria (33 Male, 86 Female) and 120 students from Poland (39 Male; 81 Female). The average age is 25.24 years for the Nigerian students and 28.10 years for the Polish students. Among Nigerian students, 28% are women, while among Polish students, 32% are women. This proportion is similar to the typical sex ratio (70% F; 30% M) at university institutions in Poland^50^.

### Procedure

To carry out the study, two identical versions of the questionnaires are prepared in two languages—Polish and English. Information about the study's goal, procedure, and possibility of withdrawing from the study at any time without consequences is also prepared in two languages. An invitation to participate in the study is directed to the group of Nigerian students with the highest scores on the English skills tests. Due to the different levels of English language skills, this selection is necessary. The students in the Polish-speaking group are Polish; therefore, no restrictions are placed on language skills. The respondents complete the questionnaires during class and do not receive any benefit from participating in the study. The study is approved by the Bioethics Committee of Warsaw Management University. The study procedure is conducted in accordance with the Declaration of Helsinki. All participants provide research information, fully understand the study’s purpose, and provide informed consent to participate in the study.

### Tools

Since the research is planned to be cross-cultural, it is necessary to select tools that would allow two linguistically distinct cultures to be studied. Given that no tools are found that are culturally tested on the Nigerian population, English-language tools are used to test a group of Nigerian students. To confirm the adequacy of this solution for each of the scales used, Cronbach's α coefficients are examined. The results do not differ from the required standards, and on most scales, the results are close to the index declared by the authors of the tools used. Tools in Polish adaptation are applied to survey Polish students. One tool is adopted during this study.

#### Obesity risk knowledge

The Obesity Risk Knowledge Scale (ORK-10) is used to assess obesity-related risk. This scale contains 10 statements describing the possible effects of obesity. For each question, the respondent can answer “True”, “False” or “Do not know”. The subject receive 1 point for each correctly answered question. The score is the sum of the obtained points. The minimum score is 0, and the maximum score is 10. To study a group of Nigerian students, we use the original English-language version developed by Swift et al.^51^. To study a group of Polish students, we use the Polish-language adaptation developed by Styk et al.^17^.

#### Attitudes and beliefs about people with obesity

In this study, we use the Attitudes Toward Obesity People Scale (ATOP), which is a 20-item tool that measures stereotypical attitudes toward people with obesity. In each question, respondents are asked to indicate the extent to which they agree or disagree with a particular statement, such as “Obese employees cannot be as successful as other employees”. Scores range from 0 to 120, where higher scores reflect more positive attitudes toward people with obesity. Beliefs about the causes of obesity are measured with the Beliefs About Obesity Scale (BAOP), which is an 8-item Likert scale that assesses beliefs about causes of obesity. In each question, individuals are asked to indicate their degree of agreement or disagreement with a specific statement about obesity causes, such as “Obesity is truly caused by a lack of willpower.” Scores range from 0 to 48, in which higher scores indicate a belief that obesity is uncontrollable. Nigerian students are surveyed with the English-language version of the scale. Polish respondents are tested with the same scales as the Polish adaptation of the Styk et al.^17^.

#### Body image

The Body Esteem Scale (BES) developed by Franzoi and Shields is used to measure satisfaction with various aspects of one's body^52^. The scale in its original English version is used for measurements in a group of Nigerian students. A Polish adaptation of the scale by Lipowska and Lipowski is used in a group of Polish students^53^. Respondents rate on a 5-point scale from *definitely dislike* to *definitely like*, the indicated body-related aspects (e.g., smell) and body parts (e.g., nose, thighs). The scale provides an overall score depicting the respondents' attitudes toward their bodies. The higher the score is, the more positive the attitude toward the body.

#### Body mass index

To determine the normality of one's body weight, body mass index (BMI) is used. This index is a simplified method for determining the normality of one's body weight. BMI is categorized as follows: underweight (BMI < 18.5 kg/m^2^), normal (BMI 18.5 ≤ 24.9 kg/m^2^), overweight (BMI 25 ≤ 30 kg/m^2^) or obese (BMI ≥ 30 kg/m^2^)^54^. BMI is calculated based on the participants’ data on height and weight.

#### Fat Phobia

In this study, we use the 14-item Fat Phobia Scale (FPh) developed by BE Robinson^55^. The scale assesses support for negative stereotypes about overweight people or people with obesity. The scale contains 14 pairs of antonyms (for example, “lazy” versus “hardworking”, “has no willpower” versus “has willpower”); participants choose one of five points on a spectrum between each word and its opposite word. The scale is designed so that higher scores indicate more negative attitudes. Cronbach’s α coefficients of the English-language scale range from 0.86 to 0.92. In the study of students from Nigeria, we use the English-language original scale version, for which we obtain a Cronbach’s α of 0.91. The scale do not have a Polish version. Therefore, we request that the authors’ permission to adapt the scale before the study began. After receiving permission, we proceed with the procedure of translating and validating the scale in accordance with the procedures we have already used earlier in translating the ATOP and BAOP scales^17^. As a result of this work, we obtain a Polish-language version of the scale (Appendix 1). We use a prepared scale in the present study. To verify whether the properties of the scale are acceptable, we perform statistical analyses on the collected group of Polish students. To verify whether the collected sample is adequate for the analyses, the KMO sample adequacy test and Bartlett's sphericity test are applied. The KMO value is 0.84, and the result of the Bartlett sphericity test is statistically significant (χ2 = 852.26, *p* < 0.001), which lead to a factor analysis. These analyses confirm a univariate factor structure, consistent with the original English-language scale, which account for 41% of the total variance. All the items had loading factors above 0.5. To assess the internal consistency of the scale, Cronbach's alpha coefficients are calculated, which yielded a value of 0.89. Therefore, we assum that the prepared Polish-language scale version meet the requirements for psychometric tools, and in further analyses, we use the results collected with it.

### Statistical analyses

The statistical analyses are performed using JASP 0.17 software. The collected data are analysed to determine which data could be subjected to parametric tests. For the data that could be evaluated using parametric tests, Student's t tests and Pearson's r correlation analysis are performed to compare the groups. For the remaining data, the Mann‒Whitney test and Spearman's rho analysis are used. The significance of differences between sample correlations is determined by Fisher's Z test using an online calculator available at www.psychometrica.de.

### Supplementary Information

Supplementary Information.

## Data Availability

All relevant data are available within the presented manuscript. Any material and information generated during the study will be available for sharing with other researchers under appropriate institutional agreements. Any inquiries should be directed to the corresponding author. The data used in the paper are available upon request.
