# Supplementary material for: Weight biases, body image and obesity risk knowledge in the groups of nursing students from Poland and Nigeria
Source: Sci Rep. 2024 Feb 22;14:4383. doi: 10.1038/s41598-024-54904-1 (PMC10883915; doi:10.1038/s41598-024-54904-1)
Supplement: Supplementary file 1 — Supplementary Information. [file 41598_2024_54904_MOESM1_ESM.docx]

**APPENDIX 1 Polish version of the Fat Phobia Scale (short form)**

Skala Fobii Tłuszczowej (F Scale) by BE Robinson w polskiej adaptacji Styk et. al.

Poniżej znajduje się lista 14 par przymiotników opisujących osoby o zwiększonej masie ciała. Dla każdej pary przymiotników wstaw znak X w wierszu najbliżej przymiotnika, który według Ciebie najlepiej opisuje Twoje odczucia i przekonania

| leniwa |  |  |  |  |  | pracowita |
| --- | --- | --- | --- | --- | --- | --- |
| bez silnej woli |  |  |  |  |  | z silną wolą |
| atrakcyjna |  |  |  |  |  | nie atrakcyjna |
| dobra samokontrola |  |  |  |  |  | niska samokontrola |
| szybka |  |  |  |  |  | wolna |
| wytrzymała |  |  |  |  |  | słaba |
| aktywna |  |  |  |  |  | nie aktywna |
| słaba |  |  |  |  |  | silna |
| samolubna |  |  |  |  |  | poświęcająca się |
| nie lubi jeść |  |  |  |  |  | lubi jeść |
| nie zgrabna |  |  |  |  |  | zgrabna |
| nie objada się |  |  |  |  |  | objada się |
| niepewna siebie |  |  |  |  |  | pewna siebie |
| z niską samoocena |  |  |  |  |  | z wysoką samooceną |

Instrukcje punktacji:

Krok 1: W przypadku pozycji 3, 4, 5, 6, 7, 10 i 12 punktacja jest następująca: 12345

Krok 2: W przypadku pozycji 1, 2, 8, 9, 11, 13 i 14 punktacja jest następująca: 54321

Krok 3: Zsumuj wynik dla każdego elementu, aby uzyskać łączny wynik.

Im wyższy wynik tym wyższy poziom fobii tłuszczowej
